# Evaluating Electroporation and Lipofectamine Approaches for Transient and Stable Transgene Expressions in Human Fibroblasts and Embryonic Stem Cells

**DOI:** 10.22074/cellj.2015.5

**Published:** 2015-10-07

**Authors:** Mehdi Sharifi Tabar, Mahdi Hesaraki, Fereshteh Esfandiari, Fazel Sahraneshin Samani, Haghighat Vakilian, Hossein Baharvand

**Affiliations:** 1 Department of Molecular Systems Biology, Cell Science Research Center, Royan Institute for Stem Cell Biology and Technology, ACECR, Tehran, Iran; 2 Department of Stem Cells and Developmental Biology, Cell Science Research Center, Royan Institute for Stem Cell Biology and Technology, ACECR, Tehran, Iran

**Keywords:** Electroporation, Lipofectamine, Genetic Modification

## Abstract

**Objective:**

Genetic modification of human embryonic stem cells (hESCs) is critical for their extensive use as a fundamental tool for cell therapy and basic research. Despite the fact that various methods such as lipofection and electroporation have been applied to transfer the gene of interest (GOI) into the target cell line, however, there are few re- ports that compare all parameters, which influence transfection efficiency. In this study, we examine all parameters that affect the efficiency of electroporation and lipofection for transient and long-term gene expression in three different cell lines to introduce the best method and determinant factor.

**Materials and Methods:**

In this experimental study, both electroporation and lipofection approaches were employed for genetic modification. pCAG-EGFP was applied for tran- sient expression of green fluorescent protein in two genetically different hESC lines, Roy- an H5 (XX) and Royan H6 (XY), as well as human foreskin fibroblasts (hFF). For long-term EGFP expression VASA and OLIG2 promoters (germ cell and motoneuron specific genes, respectively), were isolated and subsequently cloned into a pBluMAR5 plasmid backbone to drive EGFP expression. Flow cytometry analysis was performed two days after trans- fection to determine transient expression efficiency. Differentiation of drug resistant hESC colonies toward primordial germ cells (PGCs) was conducted to confirm stable integration of the transgene.

**Results:**

Transient and stable expression suggested a variable potential for different cell lines against transfection. Analysis of parameters that influenced gene transformation ef- ficiency revealed that the vector concentrations from 20-60 μg and the density of the sub- jected cells (5×10^5^and 1×10^6^cells) were not as effective as the genetic background and voltage rate. The present data indicated that in contrast to the circular form, the linearized vector generated more distinctive drug resistant colonies.

**Conclusion:**

Electroporation was an efficient tool for genetic engineering of hESCs compared to the chemical method. The genetic background of the subjected cell line for transfection seemed to be a fundamental factor in each gene delivery method. For each cell line, optimum voltage rate should be calculated as it has been shown to play a crucial role in cell death and rate of gene delivery.

## Introduction

Human embryonic stem cells (hESCs) benefit from unparalleled characteristics which introduce them as a valuable source for regenerative medicine and developmental biology (1-5). Differentiation of hESCs is challenging due to the involvement of various signaling pathways and complex gene regulatory networks in this process. Functional studies of master regulatory genes are indispensable to reach an understanding of molecular events that regulate differentiation mechanisms. To this aim, optimization of the best gene delivery approach seems to be a substantial step (6-9). Two categories have been applied for gene delivery in human and mouse ES cells -viral and non-viral. The viral method is based on a backbone derived from a viral genome, such as a retrovirus or a lentivirus that carries the gene of interest (GOI). The non-viral method is a plasmid based approach which can be used for random integration or gene targeting by a homologous recombination system (10, 11). Currently two different techniques exist that deliver the GOI into the appropriate tissue by plasmids-chemical (lipofectamine) and mechanical (electroporation). Thus far, transduction efficiencies of 20-85% have been reported for viral delivery in mouse ES cells (12). In contrast, a lower rate (1-20%) of gene transformation is reported for lipofection and electroporation (13-16). The decreased efficiency of these methods has been an important challenge; therefore, optimizing all transformation parameters will lead to a more efficient gene delivery. Electroporation and lipofectamine have been employed for transfection of human embryonic and mesenchymal stem cells with different efficiencies (17, 18). Different efficiencies (1- 30%) for random integration and gene targeting in hESCs exist (19, 20). A variety of reports that discuss gene delivery approaches via different techniques exist, however studies that have investigated all parameters of these methods in order to introduce the most efficient approach are lacking. In this study, we have modified different parameters and used different cell lines to examine the efficiency of both lipofection and electroporation. In addition, we attempted to introduce a feasible, comprehensive protocol for transient and stable transgene expression in hESC lines.

## Materials and Methods

### Vector design and construction

**Fig.1:**
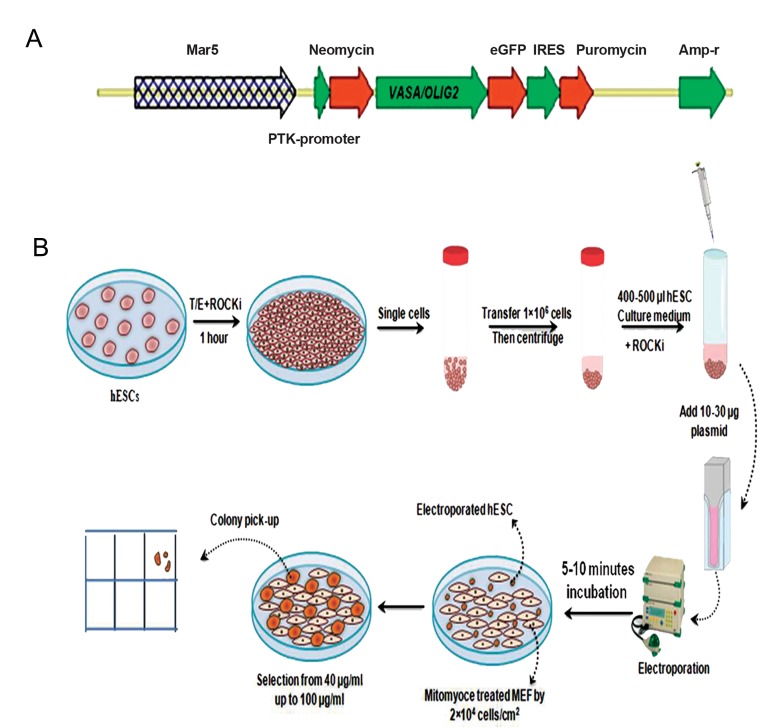
Physical map of the vectors and electroporation procedure. A. Both *pVASA*-EGFP and *pOLIG2*-EGFP benefit from dual selection system including neomycin and puromycin. No difference was seen in transformation efficiency despite the different vectors’ sizes and B. Human embryonic stem cells (hESCs) were seeded onto 50 cm plates and grown in appropriate medium until they reached 70-80% confluency. Following incubation in Tryp/LE at 37˚C for 5 minutes the cells were singled. For electroporation, the cells were counted and resuspended in phosphate buffer saline (PBS) at a concentration of 1×10^6^, after which 700 μl of cell suspension was mixed with 20-60 μg of linear plasmid DNA in a sterile electroporation cuvette. The voltage varied from 240 to 300 V. Immediately after electroporation, the cells were removed from the cuvette and plated on three 10 cm diameter tissue culture dishes in complete medium. After 48 hours, the plates were washed twice with phosphate buffer saline (PBS), then replenished with complete medium. MEF; Mouse embryonic fibroblast.

In this experimental study, we used previously described standard cloning techniques to construct the recombinant plasmids (21). Vector NTI software was used to design the specific primers for promoter isolation and quantitative real time-polymerase chain reaction (qRT-PCR) experiments (Table 1). Isolation of genomic DNA was performed using a Gentra Puregene Cell Kit (Qiagen, USA) according to the manufacturer’s instructions. Promoter isolation was conducted with a platinum Taq DNA polymerase high fidelity enzyme (Lifescience, USA) and specific primers that carried suitable restriction enzyme sites. After column purification of both the pBluMAR5 plasmid backbone and PCR products, they were subjected to restriction digestion with MluI and AgeI (Fig .1A). Following overnight digestion the fragments were gel purified and ligation performed with T4 DNA ligase. The ligation products were transformed into E. coli competent cells after which antibiotic resistant colonies were analyzed by colony PCR and restriction digestion.

**Table 1:** List of primers used for cloning and quantitative real-time polymerase chain reaction (PCR)


Amplicon name	Forward primer	Reverse primer	Length (bp)

*VASA promoter*	CCAGCCGAGTCTAACTTTC	TGGTGGCTTCAAGTTCTATTC	1534
*OLIG2 promoter*	AAATTCAGCTCGGGGAAGAG	GAAGATAGTCGTCGCAGCTTTC	2360
*EGFP CDs*	ATGGTGAGCAAGGGCGAGG	CTTGTACAGCTCGTCCATGC	720
*DAZ*	TTGCAGCAGACATGGTGGTGGC	TGTTCCAGCGGACTTCACCAGC	110
*DAZL*	TACAGGGACCAGGAGGGAACCA	CGTGGCTCCGCAAGATGGC	101
*VASA*	TTCTTGACAAAGAAAAGTTGCAATA	CGTTGAAATTCTGCGAAACA	91


### Human embryonic stem cell culture

hESC lines Royan H6 (derived from a male embryo) and Royan H5 (derived from a female embryo) (22) were cultured on Matrigel-coated plates. Cells were expanded in Dulbecco’s modified Eagle’s medium that consisted of Ham’s F-12 (DMEM-F12, Lifescience, USA, 21331- 20) supplemented with 20% knockout serum replacement (KOSR, Gibco, USA, 10828-028), 1% nonessential amino acids (NEAAs, Lifescience, 11140-035), L-glutamine (2 mM, Lifescience, 25030-024), penicillin (100 mg/ml), streptomycin (100 mg/ml, Lifescience, 15070- 063), β-mercaptoethanol (0.1 mM) and basic fibroblast growth factor (bFGF, 100 ng/ml, Royan Institute, Iran) (23). To adapt hESCs to a single condition, the cells were cultured as single cells using Tryp/LE for passage prior to the beginning of the transformation. hESCs at 75- 80% confluency were washed with phosphate buffer saline (PBS-Lifescience) and incubated with Tryp/LE enzyme (Lifescience) at 37˚C for 5 minutes. The enzyme was removed and the dissociated cells were cultured in the medium containing 10 μM rock inhibitor (Sigma, USA).

### Human foreskin fibroblast culture

Human foreskin fibroblast (hFF) cells were maintained in fibroblast medium that contained DMEM (Gibco, 12800-116), 100 U/ml penicillin, 100 μg/ml streptomycin (Gibco, 15070-063), and 10% fetal bovine serum (FBS, Gibco).

### Plasmid transformation

Plasmid transformation was carried out using lipofectamine 2000 and electroporation.

### Lipofectamine

hESCs were plated onto Matrigel and mouse embryonic fibroblast (MEF, at 50% confluency), while hFF cells were seeded on coated gelatin (0.1%). When the cells reached the appropriate confluency (70-80%), different concentrations of linearized plasmid, F12 medium and Lipofectamine® 2000 (Lifescience) were mixed according to the manufacturer’s protocol. The prepared solution was added to the cells, after which cells were incubated at 37˚C and 5% CO_2_. One day after transfection, the medium that contained plasmid was removed and replaced with approximately 3.5 ml complete culture medium. For transient expression, the cells were trypsinated and transferred into separate 5 ml flow cytometry tubes. Next, trypsinated cells were centrifuged at 200 g at room temperature for 5 minutes, after which the supernatant was discarded. In order to establish a genetically modified cell line with *pOLIG2*- EGFP and *pVASA*-EGFP vectors, the transformed cells were subjected to G418 antibiotic selection for three weeks.

### Electroporation

Electroporation is the most commonly used transformation method in hESCs by which electrical impulses that create transient pores in the cell membrane allow foreign DNA to enter into the cells. In brief, 600 μl of the previously singled cells that contained 10-60 μg linearized plasmid was transferred into the electroporation cuvette (BioRad, USA, #165-2088). Electroporation was performed in a 4 mm gap cuvette using a Gene Pulser (BioRad, Munchen, Germany) with different electric parameters that included 220 V- 500 μF, 300 V- 500 μF using one pulse (Fig .1B) (24). After pulsing, the cuvette was incubated on ice for 10 minutes. The electroporated cells were divided into two, 6 cm plates, one coated with Matrigel and the other coated with MEF. One day after electroporation the medium was refreshed by 3.5 ml complete medium. In order to study the transient expression of exogenous gene in hESCs and hFF, at 48 hours after transfection all cells were trypsinated and transferred to separate 5 ml flow cytometry tubes, then centrifuged at 200 g at room temperature for 5 minutes. The pellets were resuspended in 1 ml PBSfor EGFP expression analysis. To select stable integrants from other cells, we added 100 μg/μl of G148 to the medium. The colonies remained in the antibiotic medium.

### Transgenic stem cell pluripotency characterization

**Fig.2:**
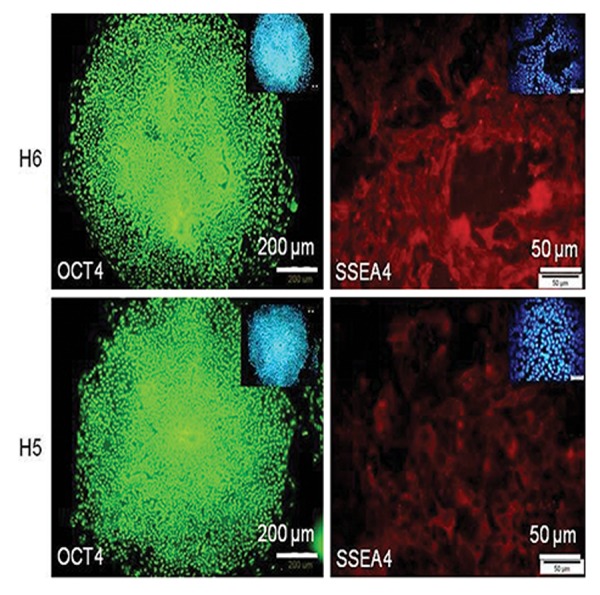
Immunofluorescence assay for stem cell markers in transgenic cell lines. Human embryonic stem cells (hESCs) were reseeded onto Matrigel-coated plates, then fluorescein isothiosyanat (FITC) and Dylight secondary antibodies were used for immunofluorescence staining of OCT-4 and SSEA-4 stem cell markers, in both H6 (A, B) and H5 (C, D) cell lines. 4΄,6-diamidino-2-phenylindole (DAPI) staining was used as a nuclear marker. Both transgenic Royan H5 and Royan H6 cell lines retained their pluripotency state.

The immunostaining assay was conducted as follows. Transgenic hESCs Royan H5 and H6 were fixed with 5% paraformaldehyde (Sigma-Aldrich, P6148) for 10 minutes, after which their membranes were permeabilized by 0.3% Triton X-100 (Sigma-Aldrich, T8532) and blocked with 10% host serum in 1% bovine serum albumin (Sigma- Aldrich, A3311). The cells were placed overnight at 4˚C with the following primary antibodies: mouse anti-SSEA4 (1:250) and mouse anti-OCT4 (1:250) diluted in blocking solution. Washing was performed three times with 0.1% Tween 20 (Sigma- Aldrich, P7949) in PBS-, and cells were incubated at 37˚C with the following secondary antibodies- goat anti-mouse fluorescein isothiosyanat (FITC) conjugated (1:200, Santa Cruz Biotechnology, sc-2010) and goat anti-mouse Dylight conjugated (1:200, Santa Cruz Bio-technology, sc-2780) for 45 minutes. Nuclei were counterstained with 4΄,6-diamidino-2-phenylindole (DAPI, 1:1000, Sigma-Aldrich, D8417) and analyzed with a fluorescence microscope (Olympus, IX71) (Fig .2).

### Stable cell line generation and colony pick-up

Three days after transformation, the cells were subjected to drug selection by the addition of G418 (geneticin) to the medium. Initially, a low concentration of the antibiotic (25 μg/ml) was used for the first few days, then the concentration was increased to 100 μg/ml. Selection with G418 (100 μg/ml) continued over two weeks in both groups. The high concentration (100 μg/ml) of G418 resulted in a wide range of cell death with no colonies visualized on the plate. Interestingly, when the plates were maintained under the same condition for an additional number of days, drug resistant colonies appeared and selection continued for two additional weeks. Antibiotic resistant colonies were handpicked from neomycin-resistant embryonic fibroblast feeder cells by a micropipette and each colony was transferred into a well of a Matrigel coated 24-well culture dish. The cells were subsequently expanded and propagated in the presence of G418. Finally, each well was divided into two parts: one part was frozen whereas the other was used for genomic DNA extraction and PCR analysis.

### Screening by polymerase chain reaction and quantitative real-time PCR

PCR analysis was carried out using EGFP specific primers (Table 1) to confirm the presence of this gene in the genome of the putative transgenic cells lines. Vector NTI was utilized to design *DAZL* and *DAZ* specific primers for qRT-PCR analysis (Table 1). Initially, total RNA was extracted using a Micro Kit (Lifescience) and whole RNA was subjected to cDNA synthesis (cDNA Synthesis Kit, Fermentas, Germany, KI632) according to the manufacturer’s instructions. Synthesized cDNA was mixed with 1x Power SYBR Green PCR Master Mix (ABI, Prism, USA, 4368702) and specific primers were added to achieve a final volume of 20 μl. We used a Corbet instrument to run the expression profiling experiment.

### Flow cytometry for transgene expression analysis

Flow cytometry analysis was performed three days after transfection. The cells were washed twice with KO-DMEM, dissociated with trypsin, then centrifuged and resuspended at 1×10^6^ cells/ml in PBS-. The cells were stored at 4˚C for a maximum of 1 hour before analysis. Acquisition was conducted on a fluorescence- activated cell sorting (FACS) Calibur system (BD Biosciences, Heidelberg, Germany) and sample analyses were carried out by CellQuest software (BD Biosciences, Heidelberg, Germany). The gating criteria for analysis of the EGFP expressing cells were set according to the level of auto-fluorescence of a non-transfected control.

### Differentiation of H6 cell line into germ cells

Differentiation of hESCs into primordial germ cells (PGCs) was conducted to confirm the stable transgenic cell lines’ functionality, pluripotency and determine whether the transgene silencing event would occur or not. Approximately, 1000 G418 resistant hESCs were cultured as hanging drops for two days in a media that contained GMEM with 15% KSR, 0.1 mM NEAA, 1 mM sodium pyruvate, 0.1 mM 2-mercaptoethanol, 100 U/ml penicillin, 0.1 mg/ml streptomycin and 2 mM L-glutamine (all from Lifescience). The media also contained bone morphogenetic protein 4 (BMP4, 500 ng/ml, R&D Systems), leukemia inhibitory factor (LIF, 20 ng/μl, Sigma), stem cell factor (SCF, 100 ng/ml, R&D Systems), BMP8b (500 ng/ml, R&D Systems) and epidermal growth factor (EGF, 50 ng/ml, Sigma). After two days, aggregates were collected in a low-cell-binding Ubottom 96-well plate (NUNC). Differentiation was carried out over 14 days and EGFP positive cells were detected by fluorescence microscope (Olympus, IX71). Cell sorting on day 14 was performed to isolate the EGFP positive cells in order to investigate germ line specific gene expression profiling.

### Statistical analysis

All *in vitro* experiments were repeated at least three times. The standard deviation and mean value were calculated using Microsoft Excel. The mean and standard deviation of cell counts were calculated. The unpaired student’s t test was used for statistical analyses. Significance levels of P<0.01 and P<0.05 were selected.

## Results

### Characterization of transgenic colonies

**Fig.3:**
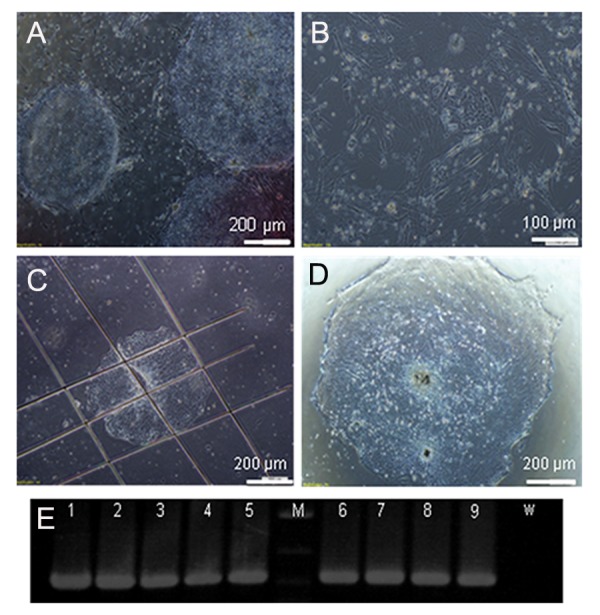
Morphology of the transgenic colonies. A. Morphology of human embryonic stem cells (hESCs) after selection with G418. Drug resistant colonies appeared after 3 days of selection, B. Lack of distinguished resistant colony formation when the cells were transformed by supercoiled plasmid, C. The antibiotic resistant colonies were picked-up by a Pasteur pipette and passaged in duplicate into wells of a 48-well tray, D. Re-establishment of a single colony that was cut from the antibiotic resistant colonies and E. PCR analysis of DNA isolated from antibiotic resistant cells using a pair of specific primers for amplification of the 720 bp EGFP gene on agarose gel. M; 1.0 kb plus DNA ladder (Gibco BRL), 1-9; Presence of EGFP in transgenic lines and W; Wild type (non-transgenic cells).

Earlier studies examined Matrigel-coated plates as an appropriate choice for seeding electroporated cells. Here, we seeded electroporated hESCs on both Matrigel and MEF to compare their impact on cell survival and stemness features (Table 2). Results indicated that both systems properly maintained the stem cells, with some difference in the number of cells that survived, as well as the shape and size of electroporated cells (Fig .3). In order to determine whether G418 resistant clones still expressed stem cell pluripotency markers, we examined expressions of several pluripotency markers by immunofluorescence staining. Expressions of Oct4 and SSEA4 were readily detected (Fig .2).

**Table 2:** Number of drug resistant colonies that appeared on mouse embryonic fibroblasts (MEF) and Matrigel after transfection


Method	Plasmids	*pOLIG2-EGFP*	*pVASA-EGFP*

Electroporation	MEF	188	195
	Matrigel	123	115
Lipofectamine	MEF	40	44
	Matrigel	19	21


Neomycin-resistant MEF cells supported the growth of transgenic human embryonic stem cells (hESCs) better than Matrigel. The numbers are the mean of three biological replicates.

### Linearized plasmid and voltage pulse affect transfection efficiency

Initially, to obtain a better view of the optimum conditions for gene transformation, we tested a range of vector concentrations, cell confluency, voltage, and linearized versus supercoiled plasmids. For transient expression, we used plasmids at a concentration range of 20-60 μg. Transformation this range of plasmids led to the appearance of an almost equal number of colonies 48 hours after electroporation (approximately 50 colonies for seeding 1×10^6^ cells) (Table 3). In addition, different cell numbers used for transfection (5×10^5^ and 1×10^6^) did not result in higher efficiency. Electroporation of the cells at a range of voltage rates (200-300 V) showed that 300 V led to more colonies (Table 3). We compared the linearized and circular plasmid transformation results in the stable cell line generation and concluded that more drug resistant colonies were generated when the plasmids were linearized (Fig .3A, B). Our data revealed that neither the plasmid concentration nor the cell number was as important as the voltage. It seemed that the circular plasmid was suitable for transient expression due to episomal expression of the transgene. On the other hand, linearizing the plasmid would be noticeable for stable transgene expression and could heighten the efficiency of the transformation.

**Table 3:** The percentage of GFP positive cells with different starting cell numbers and voltage rates


Method	Cell lines	Royan H5		Royan H6		Human foreskin fibroblast (hFF)	

Electroporation	Cell number	1×10^5^	1×10^6^	1×10^5^	1×10^6^	1×10^5^	1×10^6^
	V_200_	0.47%	0.58%	5.65%	5%	9.5%	10.57%
	V_250_	0.97%	1.1%	8.7%	9.5%	17%	18.4%
	V_300_	10.2 %	11.03%	17.2%	17. 6%	31%	29.5%
Lipofectamine		1.3%	1.5%	1.1%	0.96%	4.39%	5%


The percentages are the mean of three biological replicates. pCAG-EGFP has been used for transient expression.

### Cell line genetic background affected gene delivery rate via electroporation or lipofection

**Fig.4:**
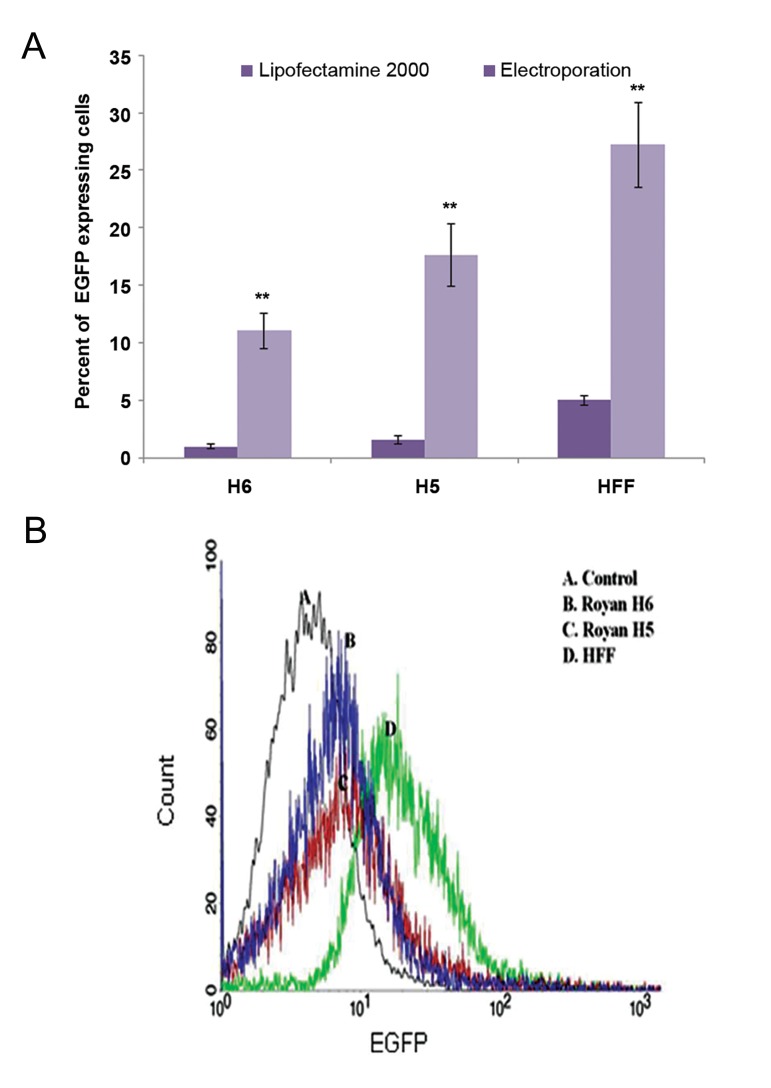
In vitro transfection of H5, H6 and human foreskin fibroblast (hFF) cell lines by lipofectamine and electroporation. A. A comparison of chemical and physical technique efficiencies for gene transformation confirmed a higher rate of transfection for electroporation in individual cell lines. The graph shows the averages of three independent experiments. Error bars represent the standard deviation. **; P<0.01 and B. Flow cytometric analysis of EGFP expression in three independent experiments. After 48 hours of gene delivery, we analyzed transient expression of EGFP by flow cytometry. hFF cells showed the highest percentage (27%) of expression when compared with the other cells. Interestingly, a comparison of the two different human embryonic stem cells (hESCs) demonstrated that Royan H5 exhibited greater transformation potential (approximately 17 vs. 10%).

A range of gene transfer efficiencies have been reported for different applied approaches and cell lines (25-27). A recent study demonstrated that responses of the H9 and H1 cell lines were not equal to plasmid transfection by either liposome based or mechanical methods. Most studies have not examined all elements involved in transformation efficiency. Here, we attempted to use the best transfection and culture conditions in order to obtain the highest number of electroporated cells (Fig .4A). For transient expression, we used the pCAG-EGFP vector followed by FACS analysis at 48 hours after transfection. The highest percentage of EGFP expression was related to electroporated hFF (~30%), whereas, among the embryonic stem cell lines H5 had a higher (17%) response to transformation. This response was almost two times more efficient than H6 (10%, Fig .4B). All examined protocols for both electroporation and lipofection approaches in hESCs had the best results for the H5 (XX) cell line. These data indicated that the genetic background of the transfected cells strongly affected the rate of gene delivery (Fig .4B, C).

### Electroporation as a suitable method for producing stable cell lines

Electroporation is the method of choice to produce transgenic mouse ES cell lines (28). However, more investigations are necessary in order to check this approach efficiency in human ESCs. Therefore, we have examined electroporation with the intent to produce genetically modified hESCs and compare them with the liposome based method. A significant difference was seen between the applied techniques (P<0.01, Fig .4). The number of antibiotic resistant colonies was counted after three weeks; on average electroporation led to the formation of approximately 200 colonies on a 6 cm plate, whereas lipofection resulted in the generation of 40 colonies on the same size plate. We began antibiotic selection three days after electroporation and 24 hours after lipofection, using a low concentration (25 μg/ ml) which was gradually increased to 100 μg/ml. Noticeably, immediate (24 hours) exposure of the transfected cells to a high concentration of antibiotic (100 μg/ml) hindered colony formation. Therefore, no antibiotic was added to the medium until the observation of small colonies on the plates. Drug selection continued for three more weeks, and the antibiotic was increased gradually to100 μg/ml. Although lipofectamine has been considered a successful approach to transient and stable cell line generation, the efficiency of this method appeared to be much lower than electroporation.

### Germ line differentiation led to *VASA* expression on day 14

**Fig.5:**
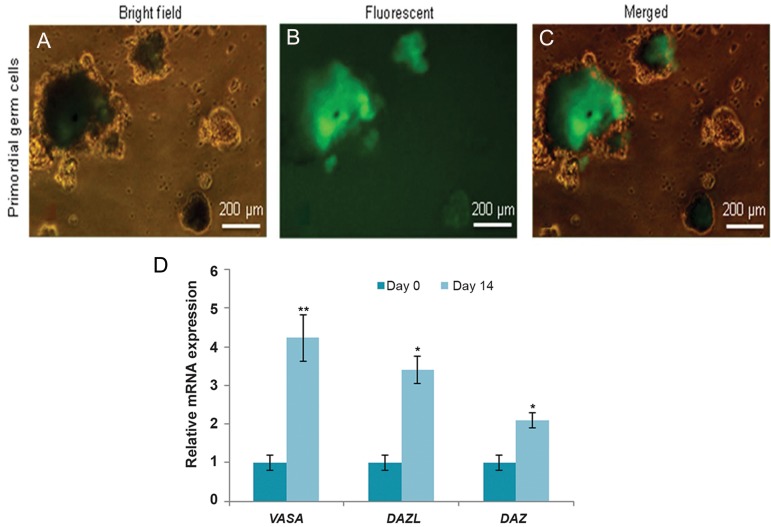
Stable expression of EGFP in primordial germ cells (PGCs) under the control of a VASA promoter. A, B, C. Bright-field, fluorescence and merged pictures of PGCs. For PGCs, the image shows the structures that arose during a 14-day differentiation time period with associated appearance of EGFP positive fluorescent cells by fluorescence microscope and D. Quantitative real-time PCR performed for expression analysis of germ cell specific genes during human embryonic stem cell (hESC) differentiation on days 0 and 14. All three germ cell specific genes significantly upregulated in EGFP positive PGCs. **; P<0.01 and *; P<0.05.

The RNA binding protein *(VASA)* is a germ line specific gene (29, 30). It is expressed in late PGCs and continues expression to spermatogonial stem cells and spermatocytes. It seems to be a reliable genetic marker to follow *in vitro* germ line differentiation. Here, we have sought to determine if the *VASA* promoter could constantly express the EGFP protein without silencing. hESCs (H6) were cultured in hanging drops in the presence of BMP4/8b, SCF, LIF and EGF for a two-day period. At two weeks after differentiation, GFP positive cells were detected within the aggregates (Fig .5) which were analyzed by FACS. Gene expression analysis at the mRNA level in sorted cells showed significant upregulation of *DAZL, DAZ* and RBMY1 compared to undifferentiated cells (P<0.01). Germ line differentiation demonstrated that genetically modified hESCs expressed transgenes during differentiation into the PGCs. Both microscopy and molecular analysis confirmed the resistance of the transgene toward silencing during germ line differentiation.

## Discussion

**Fig.6:**
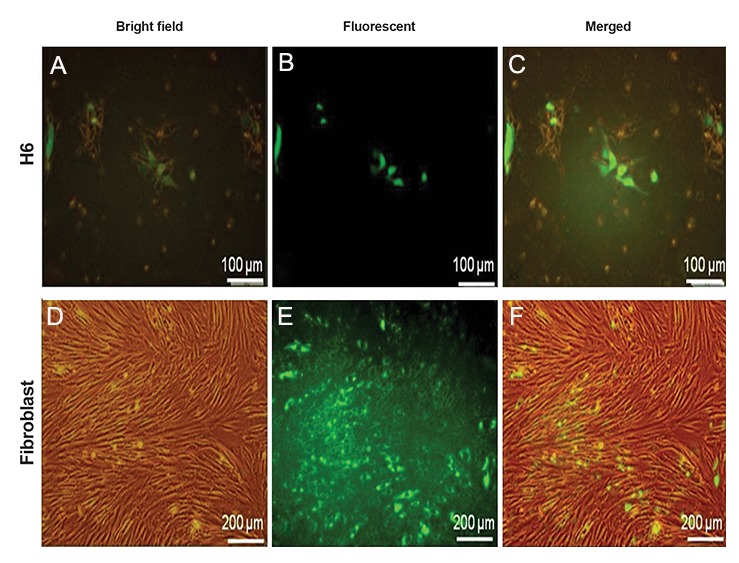
Transient expression of EGFP in Royan H6 and human foreskin fibroblast (hFF) cell lines. A, D. Bright-field images of human embryonic stem cells (hESCs) and hFF cells, B, E. Fluorescent images and C, F. Merged bright-field and fluorescent images. The pictures showed that hFF responded to electroporation more efficiently than hESCs.

Transgenic hESCs are a valuable tool for developmental biology. Different systems have been proposed to successfully generate transgenic lines, among which lentiviral transduction is proven to produce a high proportion of stable integrants in hESCs. However, its application can be hindered by the limitations of vector size and time-consuming procedures. In contrast, the non-viral method, due to its advantages of safety, ease of handling and no limit for vector size is promising for gene delivery in regenerative medicine. More recently, it has been shown that most promoters support strong transient expression, however their functions are unpredictable in long-term expression (31-33). It is presumed that expressions differ with various cell lines, transfection techniques and promoter regulatory elements. It has been reported that various promoters give variable results. For example, the CAG promoter that contained the polyoma virus mutant enhancer PyF101 element showed the strongest expression regarding transient and stable transfection, but gene silencing occurred when they used cytomegalovirus (CMV) and ubiquitin C (UbiC) promoters (31). The distinct differences between individual promoters might be due to the variation among the relevant transcription factors in ES cell lines or regulatory elements on the promoters. To prevent a gene-silencing event and obtain the highest level of transient expression, we used a pCAG-EGFP plasmid in Royan H5, Royan H6 and hFF cell lines (Fig .6). Interestingly, transformation of all cell lines by electroporation was 7to 10-fold more efficient than lipofectamine dependent on the type of the transformed line. Although after electroporation the cell survival rate was lower, the transformation efficiency was significantly higher. We examined the cell line responses to transformation. FACS analysis showed hESCs selective preference to transfection whether by mechanical or chemical methods. For long-term expression analysis, *pVASA*-EGFP and *pOLIG2*-EGFP were transformed and drug resistant colony selection performed. Unlike lipofectamine, electroporation was demonstrated to be a reliable technique to obtain a stable transgene integrant cell line. Electroporated and chemically transformed single pluripotent cells gave rise to G418 resistant colonies. However, in terms of the numbers and sizes of the colonies, we concluded that electroporation would be more promising. Lipofectamine resulted in 1.5% transient transfection levels in hESCs which might be due to the toxicity effects of lipofectamine that led to a gradual loss of transfected cells over time. Therefore, stable transfectants might not be efficiently isolated by this method compared to electroporation. Conversely, drugresistant hESC clones were successfully generated by electroporation which could be grown in culture for extended periods. Expression of a marker gene under control of a tissue specific promoter aimed to isolate a pure population of committed cells or monitor the differentiation process. Here, stable expression of EGFP under the control of a germ cell specific promoter was conducted. The transfectants were subjected to further investigation by differentiation toward PGCs. The green cells not only confirmed the authenticity of the differentiation protocol, but also demonstrated that optimal size for VASA promoter could be a 1500 bp fragment upstream of the gene. In summary, the transformation rate via electroporation depended on the genetic background and varies in different lines. The rate of the voltage in electroporation is a transformation rate-limiting factor and should be optimized for an individual cell line. Based on our experience it seemed that long-term transgene expression when linearizing the target plasmid might lead to more stable integrants than the supercoiled plasmid. Interestingly, the supercoiled plasmid showed better results for transient expression.

## Conclusion

Our results highlight the importance of the cell line against transformation and suggest that electroporation is a suitable tool for gene transformation in hESCs. Achieving the optimum voltage rate for each cell line is a crucial step in transfection. In addition, linearizing the vector for transgenic cell line establishment can lead to better results.
